# European Reference Network for Rare Vascular Diseases (VASCERN) position statement on cerebral screening in adults and children with hereditary haemorrhagic telangiectasia (HHT)

**DOI:** 10.1186/s13023-020-01386-9

**Published:** 2020-06-29

**Authors:** Omer F. Eker, Edoardo Boccardi, Ulrich Sure, Maneesh C. Patel, Saverio Alicante, Ali Alsafi, Nicola Coote, Freya Droege, Olivier Dupuis, Annette Dam Fialla, Bryony Jones, Ujwal Kariholu, Anette D. Kjeldsen, David Lefroy, Gennaro M. Lenato, Hans Jurgen Mager, Guido Manfredi, Troels H. Nielsen, Fabio Pagella, Marco C. Post, Catherine Rennie, Carlo Sabbà, Patrizia Suppressa, Pernille M. Toerring, Sara Ugolini, Elisabetta Buscarini, Sophie Dupuis-Girod, Claire L. Shovlin

**Affiliations:** 1 VASCERN HHT Reference Centre, Hospices Civils de Lyon, Lyon, France; 2 Niguarda Hospital, Milan, Italy and VASCERN HHT Reference Centre, Crema, Italy; 3 VASCERN HHT Reference Centre, Essen University Hospital, Essen, Germany; 4 VASCERN HHT Reference Centre, Imperial College Healthcare National Health Service Trust, London, UK; 5 VASCERN HHT Reference Centre, ASST Maggiore Hospital, Crema, Italy; 6 VASCERN HHT Reference Centre, Odense Universitetshospital, Syddansk Universitet, Odense, Denmark; 7 VASCERN HHT Reference Centre, “Frugoni” Internal Medicine Unit, University of Bari “A. Moro”, Policlinico, Bari Italy; 8 VASCERN HHT Reference Centre, St Antonius Ziekenhuis, Nieuwegein, Netherlands; 9 VASCERN HHT Reference Centre, University of Pavia, IRCCS Policlinico San Matteo Foundation, Pavia, Italy; 10 VASCERN HHT Reference Centre, Imperial College Healthcare National Health Service Trust, London, UK and Imperial College London, London, UK

## Abstract

Hereditary haemorrhagic telangiectasia (HHT) is a multisystemic vascular dysplasia inherited as an autosomal dominant trait. Approximately 10 % of patients have cerebral vascular malformations, a proportion being cerebral arteriovenous malformations (AVMs) and fistulae that may lead to potentially devastating consequences in case of rupture. On the other hand, detection and treatment related-risks are not negligible, and immediate. While successful treatment can be undertaken in individual cases, current data do not support the treatment of unruptured AVMs, which also present a low risk of bleeding in HHT patients. Screening for these AVMs is therefore controversial.

Structured discussions, distinctions of different cerebrovascular abnormalities commonly grouped into an “AVM” bracket, and clear guidance by neurosurgical and neurointerventional radiology colleagues enabled the European Reference Network for Rare Vascular Disorders (VASCERN-HHT) to develop the following agreed Position Statement on cerebral screening:

1) First, we emphasise that neurological symptoms suggestive of cerebral AVMs in HHT patients should be investigated as in general neurological and emergency care practice. Similarly, if an AVM is found accidentally, management approaches should rely on expert discussions on a case-by-case basis and individual risk-benefit evaluation of all therapeutic possibilities for a specific lesion.

2) The current evidence base does not favour the treatment of unruptured cerebral AVMs, and therefore cannot be used to support widespread screening of asymptomatic HHT patients.

3) Individual situations encompass a wide range of personal, cultural and clinical states. In order to enable informed patient choice, and avoid conflicting advice, particularly arising from non-neurovascular interpretations of the evidence base, we suggest that all HHT patients should have the opportunity to discuss knowingly brain screening issues with their healthcare provider.

4) Any screening discussions in asymptomatic individuals should be preceded by informed pre-test review of the latest evidence regarding preventative and therapeutic efficacies of any interventions. The possibility of harm due to detection of, or intervention on, a vascular malformation that would not have necessarily caused any consequence in later life should be stated explicitly.

We consider this nuanced Position Statement provides a helpful, evidence-based framework for informed discussions between healthcare providers and patients in an emotionally charged area.

## Main text

Hereditary haemorrhagic telangiectasia (HHT) is a multisystemic vascular dysplasia that leads to telangiectases and arteriovenous malformations (AVMs) in viscera such as the lungs, liver and brain [1] HHT is estimated to affect approximately 85,000 European citizens based on a population prevalence of 1 in 6000 [2–4]. HHT can be diagnosed through a molecular gene test identifying a pathogenic sequence variant in *ENG, ACVRL1* or *SMAD4* [1]. More usually it is diagnosed clinically by the Curaçao Criteria [5] where a patient with definite HHT will have at least 3 Criteria from spontaneous, recurrent nose bleeds; multiple mucocutaneous telangiectases at characteristic sites (lips, oral cavity, fingers, nose); visceral lesions and a first degree relative with HHT according to these criteria.

For all individuals with HHT, the VASCERN HHT Outcome Measures [6] provide an index of good practice, spanning management of epistaxis (nosebleeds); screening for iron deficiency; screening for pulmonary AVMs; antibiotic prophylaxis if pulmonary AVMs are present; and specific care in pregnancy. These five areas were selected because the available evidence was strong and considered robust to emerging new evidence for what were to be universally applicable outcome measures [6]. Additional proposed topics were not incorporated, either because the evidence base was still developing (severe bleeding; hepatic AVMs, other life periods), or because interpretation and potential application of the evidence base was too varied to capture good practice in a simple metric (e.g. cerebral AVM screening).

The complexities and controversies regarding cerebral screening continue to challenge the HHT community. Cerebrovascular malformations (CVM) affect approximately 10% of HHT patients, differing by HHT genotype, and span multiple subtypes including cerebral arteriovenous malformations (AVM), and less frequently arteriovenous fistulas (AVF), capillary telangiectases, and cavernous malformations. Patients can also have developmental venous abnormalities (DVA) and/or intracranial aneurysms [7, 8]. Haemorrhagic risks from these lesions differ markedly in the general population: AVFs, large and giant aneurysms have a very high risk of rupture; AVMs are usually in the 1–2%/year range for rate of rupture; cavernous malformations lower, and telangiectases almost zero. Currently available therapies for these CVM, and particularly AVMs including endovascular embolization, surgical resection or radiosurgery, are not trivial and carry a non-negligible risk of complications.

In order to facilitate a consensus statement, the HHT-dedicated group of the European Reference Network for Rare Vascular Diseases (VASCERN HHT) undertook 24 months of structured discussions regarding adult and paediatric screening. It first generated a brief consensus statement that was included in the 2019 Orphanet Definition of HHT [1] and is now in a position to present its full Position Statement on cerebral screening in HHT.

## Definitions

### Screening

Screening means performing an intervention on people who consider themselves well in relation to the disease that the screening relates to, and where the stated or implied purpose is either to reduce the risk of future ill-health for that individual, or, where risk cannot be altered, to give information about risk that is considered valuable [9–11]. Screening is not the same as investigating a problem or symptom, such as unexplained headaches, epilepsy, or focal neurological loss. Screening programmes of asymptomatic individuals are considered only if detailed risk-benefit evaluations suggest the detection and treatment of an asymptomatic abnormality is likely to carry overall health benefits for the patient. These considerations are recognized to centre on the degree of danger posed by particular silent lesions, the safety/tolerability of the screening method, the safety and efficacy of any treatments, and the overall potential advantage offered to the recipient in terms of better management [9–11].

As outlined by Raffle and Grey, [10] there are four possible outcomes spanning benefit, no benefit, harm and no harm (Table 1). The left-hand side assumes the balance of identifying and treating a lesion results in a better outcome than non-detection and no treatment. The lower row focuses on harm which includes, but is not limited to, counselling concept concerns, screening methodology adverse events, lesion treatment complications, and broader impacts, for example on lifestyle and insurance.

**Table 1:** General Screening Concepts

	**Benefit**	**No Benefit**
**No Harm**	Screening and treatment help. No side effects or harm	Screening and treatment do not help. No side effects or harm
**Harm**	Screening and treatment help, but side effects or harm	Screening and treatment do not help, but side effects or harm

### General cerebral screening concepts in HHT

Imaging-based screening can identify most important vascular abnormalities present in an individual. This detection is sometimes used as a proxy for a benefit of screening, but the situation is more complex.

- **Where a lesion is detected**, this does not provide any direct information that the individual will experience any complication from the abnormal vessel(s), or if it will, that the complication could be prevented. Such deductions have to be made based on evidence derived from outcomes in other individuals sharing as many characteristics as possible. Given HHT cerebral screening programmes have been in operation in many countries for decades, there is a surprising paucity of longitudinal outcome data comparing screened and unscreened populations, making it necessary to use data from the general population.
- **Where lesions are excluded,** this is usually of benefit as long as the imaging is interpreted correctly, within the limits of 1) the lesion under screening, 2) the time, and 3) the methodology of the screening. However, this information does not hold true for the statement that the individual will never newly develop a neurovascular malformation after the imaging.

### Potential neurovascular treatment options

There are no medical (conservative) treatments available for cerebral vascular malformations. Any treatment consists of either neurosurgical resection and/or endovascular embolization, and/or radiosurgery. Potentially proposed treatments depend on specific features on any neurovascular lesion.

### Cerebral vascular abnormalities

Cerebrovascular abnormalities span multiple different lesions that differ in haemorrhagic risk and preponderance (Table 2). Individuals with HHT can have a number of different cerebrovascular abnormalities, including cerebrovascular malformations (CVMs). The experience across the VASCERN HHT Centres on the prevalence and consequences of these abnormalities is summarised in Table 3.

**Table 2:** Cerebrovascular lesions in HHT patients

Lesion type	Subcategory	Haemorrhage rate (per year)	Prevalence in HHT patients	Prevalence in general population	Screening in general population?^a^
**Cerebral AVMs**	Nidal type	General population estimate 2.2% (95% CI 1.7–2.7%) [13]. Ruptured = 10% in 10% the first year after haemorrhage, then goes back to usual rate of unruptured AVMs [7, 13, 24]	~ 6.2% [8]	650/100,000 [13, 25, 26]	No
	Fistula type	Much higher risk than nidal type AVM [12, 15]	~ 1.1% but uncommon in adults [8]	< 2% of AVMs [27]	No
**Capillary malformations (telangiectases)**^**b**^		0% or exceedingly rare	2.4–61% [14, 15]	0.70% [13]	No
**Cavernous malformations**		<< 1%, but higher in deep localization [13, 28]	3.50% [8]	0.6/100,000 [29]	No
**Developmental venous anomalies**		0% or exceedingly rare [30]	12% [8, 14]	3% [31]	No
**Cerebral aneurysm**		< 1% [21]	2.1–6.8% [8, 19, 20]	Likely double reported rates of 2–3.2% (95% CI 1.9–5.2) [21, 32]^c^	No

^a^Screening does not include investigation of symptomatic patients

^b^Have been included as micro-AVMs in some studies, see text

^c^Because most meta-analysis use papers that were published before CT or MR angiography became popular and able to detect smaller lesions, the prevalence of incidental aneurysms in the general population should be higher than the reported literature rates of 2–3.2%

**Table 3:** Cerebrovascular abnormalities and consequences across the 8 VASCERN HHT Centres

	Cerebral AVM/AVF excluding microAVM, DVA, telangiectases			Any cerebral VM including AVM, AVF, microAVM, DVA, and telangiectases			Cerebral aneurysm			Cerebral haemorrhage		
Centre	All	HHT1	HHT2	All	HHT1	HHT2	All	HHT1	HHT2	All	HHT1	HHT2
Bari *Italy*	21/425 (5.0%)	16/167 (9.6%)	5/205 (2.4%)	39/425 ^**a**^ (9.2%)	29/167 (17.4%)	9/205 (4.4%)	10/425 (2.3%)	3/167 (1.8%)	6/205 (2.9%)	10/542 (1.8%)	7/197 (3.5%)	3/281 (1.1%)
Crema *Italy*				12/92 ^**b**^ (13%)						1/92 ^**b**^ (1.1%)		
Essen *Germany*	14/232 (6.0%)			21/232 (9.1%)			4/232 (1.7%)			3/232 (1.3%)		
London *UK*	0/59 ^**c**^ (0%) [20]			2/59 c (3.3%) [20]			4/59 ^**c**^ (6.8%) [20]			1/59 ^**c**^ (1.6%) [20]		
Lyon *France*	26/528 (4.9%)	16/186 (8.6%)	7/235 (3.0%)	45/528 (8.5%)	23/186 (12.3%)	15/235 (6.4%)	9/528 (1.7%)	3/186 (1.6%)	3/235 (1.3%)	13/1742 (0.75%)	6/639 (1.6%)	4/ 616 (0.6%)
Nieuwegein *Netherlands*					13/157 (8.3%) [33]	1/177 (0.6%) [33]	0/196 (0%) [34]			3/196 (1.5%) [34]		
Odense *Denmark*							2/588 (0.3%)	2/285 (0.7%)	0/225 (0%)	8/588 ^**d**^ (1.4%)	7/285 (2.5%)	1/225 (0.4%)
Pavia *Italy*	14/141 (9.9%)	9/35 (25.7%)	3/46 (6.5%)	18/141 (12.8%)	10/35 (28.6%)	4/46 (8.7%)	2/141 (1.4%)	0/35 (0%)	2/46 (4.3%)	1/141 (0.7%)	1/35 (2.9%)	0/46 (0%)
Mean **%**^**e**^	6.33%			9.28%			1.43%			1.09%		
HHT1:HHT2 ^**e**^	3.42			3.15			0.63			3.30		

All VASCERN HHT Health Care Providers (HCPs, Centres) as of 26.02.2020, within Italy, Germany, the UK, France, the Netherlands and Denmark.

^a^DVAs not included

^b^Cohort of consecutive patients screened for CAVMs with MR between 2008 and 2009; after these data and on the basis of patients’ advocacy opinion, in 2009 the HCP stopped CAVM screening

^c^Excluded any cases with symptoms of concern considered to require specific investigation e.g. unexplained epilepsy, severe headaches (not migraines), and/or current focal neurological symptoms (as detailed further in [20])

^d^The total number of HHT patients also included 78 HHT patient with either a *SMAD4* pathogenic variant, or no pathogenic variant identified

^e^Calculated after summing all lesions, and all denominators in the respective columns

#### Malformations

##### i) Cerebral arteriovenous malformations (AVM)

are high flow CVMs with arteriovenous shunting connecting cerebral arteries and veins, and bypassing the capillary bed. They consist of two subtypes:

- **nidal AVMs** which consist of a tangle (or nidus) of vascular malformative connections between cerebral arteries and veins. They represent the vast majority of the cerebral AVMs in adult HHT patients, but are not seen in neonates and are extremely rare in early childhood.
- **arteriovenous fistulae (AVF**, also known as **non-nidal AVM)** are high flow CVMs which are direct communications between cerebral arteries and veins (i.e., without any nidus between the feeding artery and draining vein). They carry a particularly high haemorrhagic rate, and usually present with symptoms due to a bleed [12]. They are present in neonates, and are considered the likely reason why haemorrhagic rates are higher in childhood.

##### ii) Capillary malformations

are non-shunting low flow CVMs composed of dilated capillaries interspersed with a normal brain parenchyma with a thin endothelial lining, but no vascular smooth muscle or elastic fibre lining. They are often detected incidentally and are considered benign CVM. Apart from rare case reports, haemorrhage from capillary malformations (telangiectases) was not seen in any of the observational studies reported to date [13–15].

Some HHT CVM studies have included these lesions as “micro AVMs.” “Micro AVMs” was a term invented by the father of modern micro neurosurgery (Yasargil) to define quite small AVMs (below 1 cm), for practical surgical purposes (ease of resection). Capillary malformations are not AVMs, do not tend to bleed, and studies which include these under the umbrella of “AVMs” for prevalence and haemorrhage rate calculations tend to report higher AVM prevalence and lower overall AVM bleeding rates [7, 8, 16] than studies which exclude them, as is our preference.

##### iii) Cavernous malformations

(formerly also known as “cavernomas”) are non-shunting low-pressure angiographically occult lesions, composed of blood-filled sinusoidal locules known as “caverns.” They may rupture and lead to intracranial bleeding. However, the annual rate of haemorrhage for incidental and unruptured cavernous malformation is very low (< 0.6%) [17, 18].

##### iv) Developmental venous anomalies (DVA)

are non-shunting congenital variants of the cerebral venous drainage composed of dilated centripetally and radially oriented draining medullary veins and merge into a single collecting transcerebral vein that opens into either superficial subcortical veins or subependymal veins. These are considered as benign and asymptomatic vascular malformations.

#### Aneurysms

Aneurysms are focal dilatations of cerebral arteries and occur in HHT at a similar frequency to in the general population, so are therefore probably not HHT-related [8, 19–21]. The prevalence of incidental aneurysms in the general population should be higher than the reported literature rates of 2–3.2% (Table 2) because the smaller ones are difficult to identify and because most meta-analysis use papers that were published long before CT or MR angiography became popular. We estimate the prevalence as double this i.e. circa 4–6%. Importantly cerebral aneurysms not related to HHT-specific AVMs were the cause of a significant proportion of cerebral haemorrhages in three HHT cohorts [12, 22, 23], indicating that all cerebral haemorrhages in HHT should not be ascribed exclusively to HHT-related arteriovenous malformations (AVMs). Allowing for differences in methodologies, current estimates are that the prevalence of intracranial berry aneurysms in HHT patients is similar to that in non-HHT patients [8, 19–21].

The published data on the prevalence of these vascular abnormalities in HHT and non-HHT patients, as well as their respective bleeding risks and screening management in the general population, are reported in Table 2. Note that the rarity of the lesions and haemorrhagic events means that confidence intervals for the figures presented may exceed 25% (e.g. [7]), making comparison of small percentages problematic. There are also several biases that the reader should be aware of, the most important of which are:

i) whether the population was an “allcomer” screening programme or whether symptomatic patients were more likely to be included in the study denominator, thus increasing the likelihood of finding lesions associated with symptoms and/or haemorrhage risk (eg [12]);
ii) whether capillary telangiectases (as micro AVMs) and/or DVAs were included in a general “cerebral AVM” classification, thereby increasing the prevalence but reducing the apparent haemorrhagic rate of cerebral AVMs.

## Proposed position statements

### 1) Neurological symptoms suggestive of cerebral AVMs in HHT patients should be investigated as in general neurological and emergency care practice. This is separate to screening discussions. Management approaches should rely on expert discussions on a case-by-case basis and individual risk-benefit evaluation of all therapeutic possibilities for a specific lesion (neurosurgery, embolization, stereotactic radiosurgery).

1.1 In HHT, cerebrovascular malformations result in varying degrees of haemorrhagic risk, and they may also cause neurological symptoms related to their size or location (estimated to occur in up to 50% of the AVMs), such as headache, focal neurologic deficit, or seizures.
1.2 Even if the HHT practitioner does not suspect HHT as a cause of the symptoms, if cerebral imaging is likely to be performed or considered, then it may be useful for the patients to have an outline of the HHT related pre-screening discussions so that they are better informed if a lesion is detected. Individual cases will differ in haemorrhagic risks and need careful case-by-case assessment if detected. For example, it is accepted that cerebral AVF and other childhood lesions are more likely to haemorrhage, and paediatric screening is specifically addressed below.
1.3 If an AVM is found accidentally, management approaches should rely on expert discussions on a case-by-case basis and individual risk-benefit evaluation of all therapeutic possibilities for a specific lesion [35]. Details are deferred to expert neurovascular networks such as that being developed within VASCERN. Where there is a haemorrhagic risk of 1–2% per year, and the treatment risk in selected young cases (long life expectancy) may be much lower (around 4–5%), then treatment may be considered

### 2) The current evidence base does not favour the treatment of unruptured cerebral AVMs, [35] and therefore cannot be used to support widespread screening of asymptomatic patients.

2.1 The cerebral vascular malformations at higher risk of bleeding are those found in newborns (AV fistulas or giant aneurysms). Newborn and paediatric issues are discussed separately below.
2.2 Nidal AVMs have a lower haemorrhagic risk than AVFs, though this is still estimated (in series not confounded by inclusion of capillary telangiectasia and DVAs), as being in the order of 2% per annum. After a CAVM has ruptured, the re-haemorrhage rate is considered at 10% in the first year after haemorrhage, then it goes back to the usual rate of unruptured AVMs. So, it is of surprisingly little relevance to know whether an AVM has ever ruptured in life.
2.3 Treatment risks are not negligible. For example, in the general population, the ARUBA trial (A Randomized trial of Unruptured Brain AVM therapy) randomly assigned patients with a cerebral AVM to receive or not receive an interventional procedure (surgery, embolization, stereotactic radiotherapy) in addition to standard medical therapy [36]. The trial was halted early by the data and safety monitoring board because of a threefold increased risk of adverse outcomes in the intervention group. At a median follow-up of 33 months, the primary composite endpoint of death or symptomatic stroke was seen in 35 of 114 patients in the intervention group (31%) versus 11 of 109 patients in the medical management group (10%). Individuals in the intervention group also had worse functional outcomes [36]. It is important to note that the ARUBA trial has been widely criticized [35], and that its conclusions are valid only for a certain subtype of the AVM population, for a certain type of treatment and only for a limited life-span. Furthermore, the main goal of treatment should be to achieve full AVM occlusion (since partial occlusion does not reduce but increases the haemorrhage risk [35]), and a complete occlusion was achieved only in a small minority of patients in the ARUBA trial.
2.4 The ARUBA trial patients were not known to have HHT. HHT is estimated to be present in 2% of cerebral AVM cases [37, 38]. The study therefore is likely to have contained very few, if any, patient with HHT, and in this sense the ARUBA trial cannot be applied to them. However, there is no evidence that natural history or treatment risks are higher in cerebral AVMs associated with HHT than in the general population.
2.5 These data were summarised across the European Reference Network, that “Usually, cerebral AVMs that have not bled are not treated, whereas cerebral AVMs that have already bled or have become symptomatic usually require treatment [1]”.
2.6 Across the eight HHT VASCERN HHT healthcare providers (HCPs) in Europe, only one routinely performs cerebral MRIs to screen asymptomatic adults for cerebral AVMs. One centre’s screening practice, providing formal pretest counselling that differs according to family history is described elsewhere: [20] At this centre, across 603 HHT patients with no neurological symptoms of concern, post ARUBA screening MRI scan uptake was 11.3% (68.8% in patients with a family history of cerebral haemorrhage and 4.9% in patients with no family history of haemorrhage) [20].

### 3) Individual situations encompass a wide range of personal and clinical states. In order to avoid conflicting advice, particularly arising from non-neurovascular interpretations of the evidence base, we suggest that all HHT patients should have the opportunity to discuss knowingly brain screening issues with their healthcare provider.

Although the available evidence does not support risk reduction by post screening treatment, the stated or implied purpose of screening can be that where risk cannot be altered, it allows information considered valuable about the risk to be provided [9, 10].

3.1 Individuals receiving a negative scan result may benefit, particularly if previously anxious. However, later in life, these individuals may develop lesions, a phenomenon that is increasingly recognised in the general population (e.g. normal MRI scans up to 15–30+ years of life and later developing an AVM). Therefore a negative state cannot be secured for a lifetime, resulting in potential rescreens in later life.
3.2 Conversely, individuals receiving a positive result can face very challenging issues regarding communication of expectations, treatment programme duration and limitations. Lifestyle adjustments have been presented from the patient’s perspective [39], where they had already had a haemorrhagic stroke and were therefore in a higher-risk group for a future bleed.
3.3 Although once patients with symptoms are removed, absolute rates of cerebral AVMs may be relatively low, [7, 20] other positive findings should be expected at usual population rates (e.g., cerebral aneurysms and other neurological pathologies), or higher than usual population rates due to HHT pulmonary AVMs (e.g. infarcts as discussed further in [20]).
3.4 There is some evidence from HHT populations that the degree of anxiety differs according to pre-test information and exposure to other individuals:
  In 603 HHT patients undergoing pre-screening counselling at one VASCERN HCP, there was an increase in scan rates after communication of the ARUBA trial results which may have been expected to reduce screening uptake [20]. The interpretation was that the extra reassurance offered in case an AVM was detected may have enabled some patients to take that risk when seeking a screening scan that could exclude more worrying intracranial pathology [20].
  In the same study, there was a 14-fold higher uptake of screening scans in people with a family history of cerebral haemorrhage than those without such a family history [20].
  In a similar manner, noting cerebral AVMs are more common in HHT1 than HHT2 (Table 3, [40–45]), another HCP observed that the rate of patients accepting MRI screening was higher in HHT1 than HHT2 [44, 45].

### 4) Any screening discussions in asymptomatic individuals should be preceded by informed pre-test review of the latest evidence regarding preventative and therapeutic efficacies of any interventions. The possibility of harm due to detection or intervention on a vascular malformation that would not have necessarily caused any consequence in later life should be stated explicitly.

4.1 As mentioned above, in HHT population, the risk of haemorrhagic complication across all cerebrovascular malformations is very low and estimated of < 1% per year. Although the prevalence of cerebral AVMs is higher than in the general population, in HHT patients the unruptured AVMs may present a lower risk of rupture [7, 8]. (Based on the allocation of capillary telangiectases “micro AVMs”, and DVAs to some of these studies, it can be estimated that this may rise to the general population rate of ~ 2% per year if only cerebral AVMs are considered). Currently available therapies for AVMs including endovascular embolization, surgical resection or radiosurgery are not trivial and carry a non-negligible risk of complications.

## Paediatric considerations by VASCERN HHT

VASCERN HHT specifically evaluated if there needed to be a separate statement to span Paediatric screening. As noted above, the cerebral vascular malformations at higher risk of bleeding are those found in newborns (AV fistulas or giant aneurysms). For the neonatal period, there is little literature. It is evident that individual haemorrhagic events are rare, and that extra data is required to evaluate the additional risk posed to the offspring of an HHT-affected parent as opposed to general population neonates. All VASCERN HCPs considered CAVM-related bleeding in newborns in HHT families a priority for inclusion in the new VASCERN prospective HHT Registry in set up**.**

**Fig. 1:**
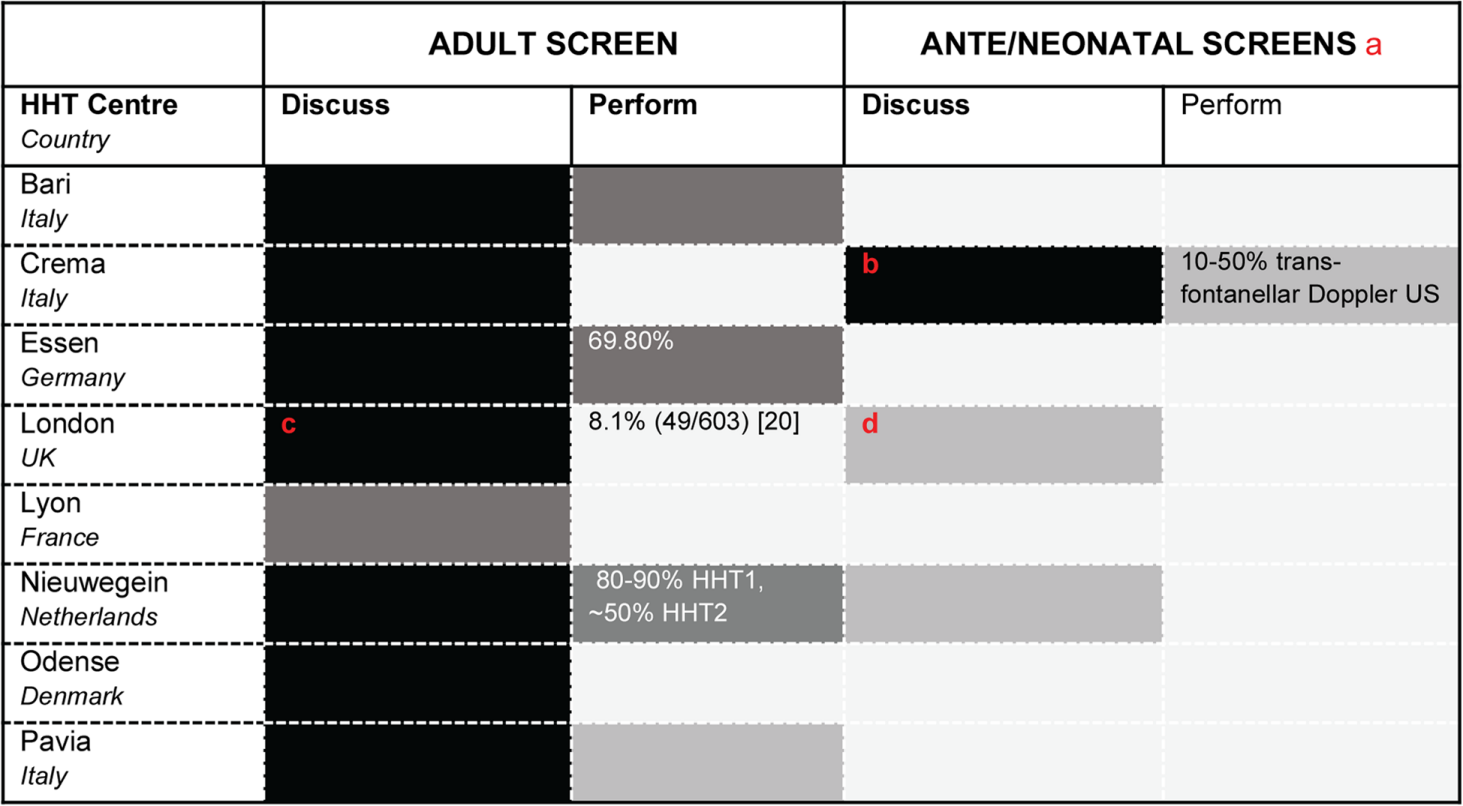
Current screening practices across the eight VASCERN HHT Centres. Proportions of the cohorts where screening is discussed and performed. 4 colour codes represent 4 broad percentage ranges: Black 90–100%; dark grey 50–90% (adult columns 1 and 2); mid grey 10–50%, light grey < 10%. **a** in addition to population-wide, country specific antenatal screening programmes. **b** Pregnancies are an indication for antenatal screening and transfontanellar Doppler US in perinatal period; MR is discussed on the basis of familial history. **c** as per protocol in [20]. **d** In any setting, discuss first, and have an open door policy for imaging if things change. Aiming too for prenatal scan support. Under 3 months, as no general anaesthetic required for MR, use “feed and wrap”

Based on available data, we examined our practices across the HCPs (Fig. 1), and concluded that the broad statements are currently also applicable to the Paediatric populations. However, the implementation varied. Notably several VASCERN HHT centres offer newborn screening, with ultrasound screening for CAVMs as an adjunct to general prenatal screens, or in the perinatal period with a transfontanellar study. MR is a very good second choice when easily available. Since our 2018 discussions, new data on general paediatric risks have become available and support the broad conclusions in general paediatrics (US, personal communication).

## Discussion/conclusion

Given the paucity of longitudinal studies regarding natural history of cerebral AVMs in HHT, the discussion aims to convey more accurate, complete and updated information to the patient by the health care provider, in order to ensure the correct patient empowerment in the final decision to accept/decline instrumental screening.

When generating a consensus statement for cerebral AVM screening in HHT, we found it important to distinguish different cerebrovascular abnormalities commonly grouped into a single “AVM” bracket, and to follow clear guidance by neurosurgical and neurointerventional radiology colleagues.

The cerebral vascular abnormalities that carry the highest risk in HHT are large and giant aneurysms on one side and high-flow AV fistulas on the other side. Both these lesions occur much more frequently (almost exclusively, probably because of the low survival rate) in the neonatal population. Most of these lesions, due to their large size, might be observed/found/screened with a simple head ultrasound echography in the newborn early days, or even just before birth.

Cerebral AVMs are a frequent finding in the adult HHT population, but they carry a lower haemorrhagic risk compared to the previous ones. In asymptomatic adult HHT patients, no screening is recommended. However, if one is found incidentally, as in general population an interventional attitude may be of value on a case-by-case base experts’ discussion, when the treatment risk is considered low (below 5–6%), because the annual risk of hemorrhage is not minimal (e.g. 2–3% per year). Similar considerations, albeit more cautiously, may apply to symptomatic cavernous malformations (cavernomas), that had bled at least once.

On the other hand cerebral aneurysms are as frequent in HHT patients as in the general population. When they are small and incidental (the great majority), they have a very low rupture risk, so that both the screening and the treatment are not recommended. The definition of “small” may vary following different authors, mainly a critical cutoff of 7 mm is used in the literature.

The position of the ERN will be revisited in 3–5 years, as new data become available.

## Methodology

In order to develop this position statement:

- Data on adult practice was collected during VASCERN Outcomes Manuscript [6] discussions, and discussed further at the October 2018 face to face meeting in order to generate very brief comments that could be approved by all for incorporation within the 2019 Orphanet text revisions [1].
- Data on current paediatric practice was collected across the eight VASCERN centers and presented at the October 2018 face to face meeting in order to generate brief consensus comments that were incorporated within the 2019 Orphanet text revisions [1].
- All groups continued to collect and generate evidence regarding their own practice including adult cerebral AVM screening protocols [20].
- Formal cerebral AVM-specific education was provided through a one-hour lecture by Essen HCP Lead Ulrich Sure at the Face to Face Meeting of VASCERN HHT in November 2019.
- Drafts were developed by CLS, SDG and US before circulation to EOF, EB and MCPa for detailed neurovascular text section writing. EOF, EB, US and MCP defined the final core messages. Subsequently, the neurovascular-approved texts was circulated for discussion across all who had been involved in discussions in the preceding two years, and additional VASCERN HHT HCP neurovascular experts for further discussion and revision. CLS coordinated comments. All authors approved the final manuscript.

## Data Availability

The datasets analysed during the current study are available from the corresponding authors on reasonable request.
